# Structural Modifications and Biological Activities of Natural α- and β-Cembrenediol: A Comprehensive Review

**DOI:** 10.3390/ph15050601

**Published:** 2022-05-13

**Authors:** Kuo Xu, Xinying Du, Xia Ren, XiuXue Li, Hui Li, Xianjun Fu, Xiaoyi Wei

**Affiliations:** 1 Shandong University of Traditional Chinese Medicine, Jinan 250355, China; xukuoworld@126.com (K.X.); dxy13064056052@163.com (X.D.); 1989renxia@163.com (X.R.); lucy_278@163.com (X.L.); lihui_210@126.com (H.L.); 2 Qingdao Academy of Traditional Chinese Medicine, Shandong University of Traditional Chinese Medicine, Qingdao 266114, China; 3 Key Laboratory of Plant Resources Conservation and Sustainable Utilization, South China Botanical Garden, Chinese Academy of Sciences, Guangzhou 510650, China

**Keywords:** *Nicotiana tabacum*, tobacco, cembrenediol, cembranoid

## Abstract

As one of the most characteristic ingredients of glandular trichome secretions from *Nicotiana tabacum* L. (tobacco), natural cembrenediols, namely, (1*S*,2*E*,4*S*,6*R*,7*E*,11*E*)-2,7,11-cembratriene-4,6-diol (α-cembrenediol/α-CBD) and its C-4 epimer (β-cembrenediol/β-CBD), have attracted considerable attention for their potent antitumor, neuroprotective, antimicrobial, and other activities. Many researchers are committed to exploring the possibility of utilizing these two cembrenediols and their derivatives both in human medicine and in agricultural fungicides. To the best of our knowledge, this review is the first to provide a comprehensive summary of the chemical modifications and bioactivities of α- and β-CBD from their discovery to the present day; the review highlights their potential medicinal value for humans. The extensive references from 1962 to 2022 provided herein were systematically gathered from the SciFinder, Web of Science, and Google Scholar databases. We expect this review to assist in providing practical ideas for future drug development based on α- and β-CBD and in further facilitating the utilization of the tobacco cembrenediols.

## 1. Introduction

**Figure 1:**
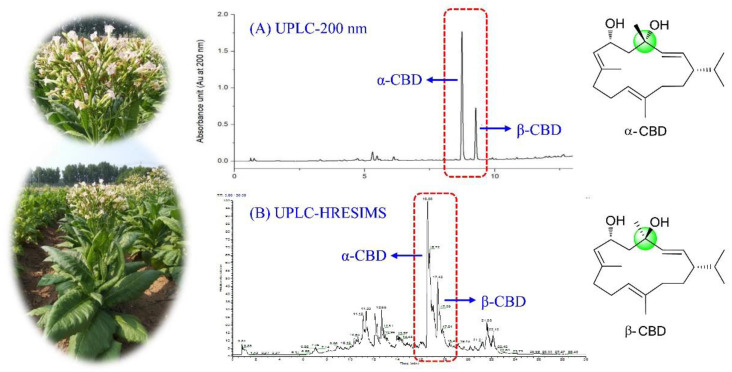
UPLC (**A**) and HPLC-HRESIMS (**B**) analyses of α-CBD and β-CBD in tobacco glandular trichome secretions.

Tobacco (*Nicotiana tabacum* L.) is an important industrial crop cultivated worldwide which generates a large amount of tax revenues for governments and increases the incomes of farmers and cigarette companies. In addition to being the raw material for the production of cigarettes, the crude extract of *N. tabacum* is used as an insecticide in folk medicine [1,2,3]. Hundreds of compounds that contain alkaloids, terpenoids, and sugar esters have been identified from this plant in bioassay-guided separation processes or other methods [4]. Among these compounds, nicotine-type alkaloids and cembrane-type diterpenoids are the two most characteristic classes from *N. tabacum*. In contrast to the alkaloids stored in plant cells, cembranoids are present as glandular trichomes on the surface of tobacco leaves and flowers; at these locations, cembranoids coincide with fatty alcohols and sucrose esters. The first cembranoids to be isolated were (1*S*,2*E*,4*S*,6*R*,7*E*,11*E*)-2,7,11-cembratriene-4,6-diol (called α-cembrenediol, or α-CBD) and its C-4R epimer (β-cembrenediol, or β-CBD), which are the major cembrenediols and the main precursors of other cembranoids from tobacco [5,6]. As shown in Figure 1, α- and β-CBD occupy a large proportion of tobacco glandular hair secretions per both UPLC and HPLC-HRESIMS detection. Both the X-ray crystal structures and the 2D NMR spectra of these compounds were first reported in the 1980s [7]. At about the same time, their structures were obtained in total synthesis experiments [8,9,10]. Cembrenediols from the glandular trichome secretions of tobacco were obtained with high yields and exhibited promising activities, making them a particularly interesting option for structural modification experiments [11]. A series of scientific investigations have indicated that α- and β-CBD could provide potential health benefits in treating cancer and neurological injuries, and these compounds have been registered with international patents in many countries. Therefore, exploiting the pharmaceutical value of α-/β-CBD and their synthetic analogs could greatly facilitate the utilization of tobacco cembrenediols.

Several excellent reviews on the related issue of tobacco cembranoids have been published over the past twenty years. As early as 2007, K.A. El-Sayed et al. systematically summarized the biocatalytic and semisynthetic studies on anticancer cembrenediols [12]. N. Yan et al. performed a review summarizing a total of 87 naturally occurring tobacco cembranoids, seven biological synthetic derivatives, and twenty-five chemical synthetic derivatives of α- or β-cembrenediol [13]. Another review that focused on the bioactivities of α- or β-cembrenediol, including against phytopathogenic fungi and insects, was published three years later [14]. At almost the same time, P.F. Yang et al. performed a systematic review of structural and biological studies on natural tobacco cembranoids [15]. Our research examining the latest studies on tobacco cembranoids revealed that the previously published reviews are incomplete, as several excellent recent studies are not included in the aforementioned reviews. The present review therefore focuses on both updating the latest scientific investigations and more importantly on providing the most comprehensive possible review of structural modifications to α- and β-CBD from the perspective of medicinal chemistry. In addition, this review provides a discussion of the pharmacological activities associated with the structural features (the structure–activity relationship, or SAR) of these compounds. An effective literature search strategy based on the SciFinder Scholar and Web of Science databases was performed in order to ensure that any related references were not overlooked. We expect this critical review to be beneficial to the development α- and β-CBD as potential raw materials for the pharmaceutical industry in the future.

## 2. Method of Searching Literature

The process of searching the relevant literature and organizing the core information into figures or tables can greatly aid authors in developing a deeper understanding of material; such a process can directly influence the final conclusions and perspectives reached [16,17]. The literature search performed in the present review utilized two prominent research discovery applications, namely, Chemical Abstracts (CA) and the Web of Science (WOS). The detailed search strategy utilized in the present review is described below. The CA database was accessed through SciFinder Scholar (Qingdao Agricultural University Library, Qingdao, China). First, the structures of α-CBD (CAS no. 1026661-36-8) and β-CBD (CAS no. 122053-29-6) were drawn using the tools in SciFinder’s drawing editor and then placed into the CA database in order to conduct a reaction and patent search. The compounds were set as ‘Material’ or ‘Reagent’ during this process. Additionally, these two compounds were placed into the WOS database (Shandong University of Traditional Chinese Medicine, Jinan, China) in order to search for relevant literature that had been previously reported on this topic. The topics in the search were ‘cembrenediol’ or ‘tobacco cembranoid’, the search was refined by ‘Document types (article)’, the timespan was ‘All years (1910–2022)’, and the indexes were ‘SCI-EXPANDED’ and ‘CPCI-S’. We used Google Scholar to survey other journals in the field of medicinal chemistry that may have been excluded from the above databases. Based on the aforementioned approach, a total of 85 reactions, 10 patents, and 45 references were ultimately collected, covering most of the related studies.

## 3. Structural Modifications of α- and β-CBD

### 3.1. Chemical Synthesis

**Scheme 1:**
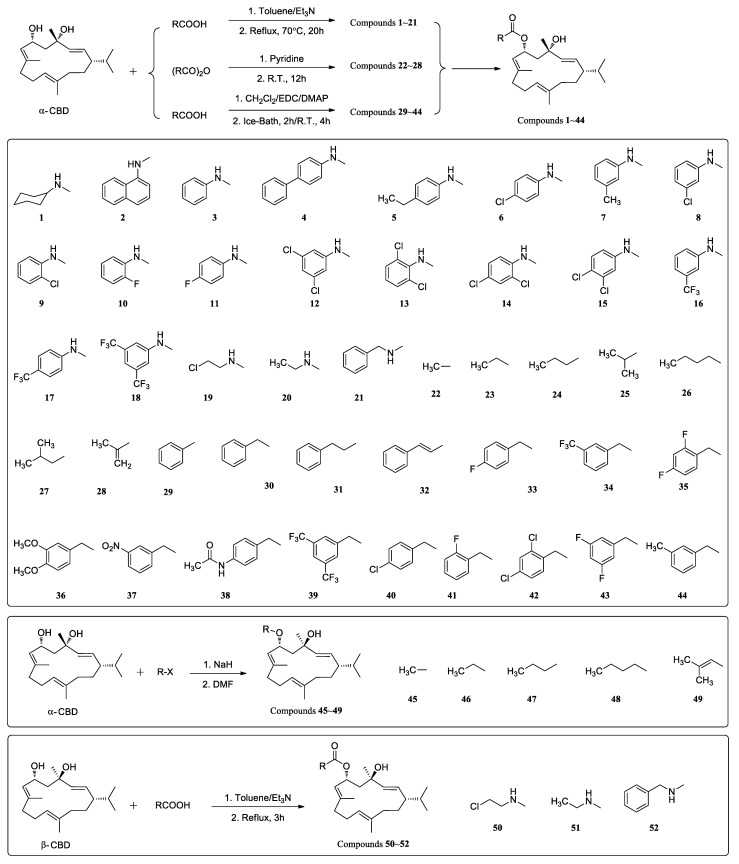
Synthesis of the 4-*O*-ester derivatives of α-CBD (**1**–**44**), β-CBD (**50**–**52**), and 4-O-ether derivatives of α-CBD (**45**–**49**).

α-CBD and β-CBD were first found to exhibit antitumor activity in 1985 [18], although little was understood regarding the structure–activity relationships of tobacco cembranoids until the research team of Professor Khalid A. El Sayed performed their pioneering work. As early as 2008 they had prepared various cembrenediol C6-ester derivatives using semisynthetic approaches. Refluxing α-CBD or β-CBD in toluene with chlororethyl, ethyl, and benzyl isocyantes and then adding a catalytic amount of triethylamine yielded the C-6 carbamates α-CBD (**19**–**21**) and β-CBD (**50**–**52**), respectively. These cembrenediol C6-ester derivatives were assayed against the proliferation of human mammary epithelial cells (HMECs) and human metastatic prostate cells (PC-3M) [19,20]. A series of α-CBD analogs (**1**–**18**) were rationally synthesized in order to explore additional targeted c-Met interactions in MDA-MB-231 cells and improve cellular potency. Compound **3** exhibited an improved c-Met inhibitory effect compared to that of α-CBD and β-CBD. The SARs of different substituents on **3′**s phenyl moiety (**3**–**18**) are discussed below [21]. A research team led by professor Duo-Bin Mao at Zhengzhou University of Light Industry designed and synthesized various cembrenediol C6-ester derivatives [22]. Twenty-eight α-CBD derivatives were prepared by modifying the C-6 position via the introduction of various acyl and ether groups. Compounds **22**–**28** were obtained via the acid anhydride–pyridine reaction system at room temperature, while compounds **29**–**44** were synthesized by an acid–base condensation reaction using the raw materials EDC [1-(3-dimethylaminopropyl)-3-ethylcarbodiimide hydrochloride] and DMAP (4-dimethylaminopyridine). The synthetic routes and structures of **1**–**52** are shown in Scheme 1.

**Scheme 2:**
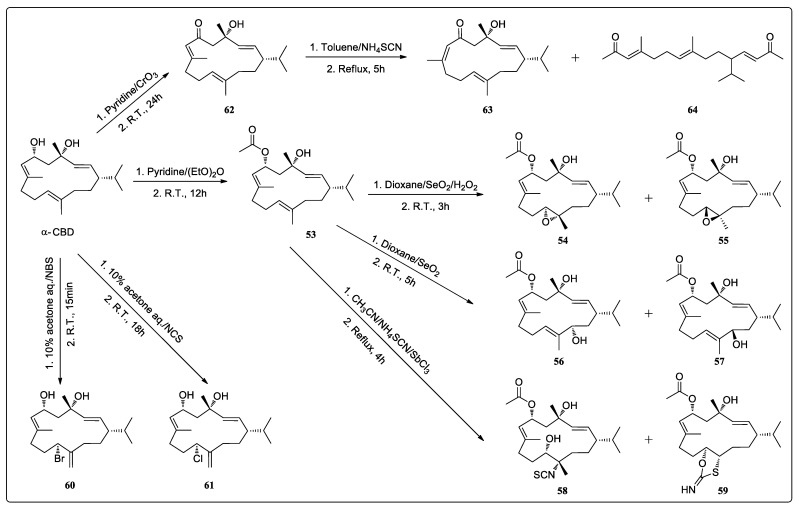
Semisynthetic acetylation, oxidation, epoxidation, and halogenation of α-CBD (**53**–**64**).

Except for the above cembrenediol C6-ester derivatives, structural modifications exist at other sites of various derivatives (**53**–**64**) [19]. Compound **53** was prepared from the acetylation of α-CBD and subsequently used as a material for different oxidation reactions. The reaction of **53**, SeO_2_, and 30% H_2_O_2_ in a 1,4-dioxane solution at room temperature afforded 11,12-epoxide analogs **54** (11S,12S) and **55** (11R,12R), while the reaction of **53** with SeO_2_ in a 1,4-dioxane solution without H_2_O_2_ yielded **56** and **57**. β-Hydroxy thiocyanate (**58**) and 1,3-oxathiolan-2-imine (**59**) were synthesized via treatment of **53** with NH_4_SCN/SbCl_3_. Halogenated derivatives **60** and **61** were prepared from α-CBD using NBS (N-bromosuccinimide) or NCS (N-chlorosuccinimide) in 10% aqueous acetone at room temperature, respectively. Refluxing α-CBD overnight with CrO_3_ in pyridine afforded **62**, which was then treated with NH_4_SCN in toluene solution to yield **63** and **64**. The synthetic routes and structures of **53**–**64** are shown in Scheme 2.

**Scheme 3:**
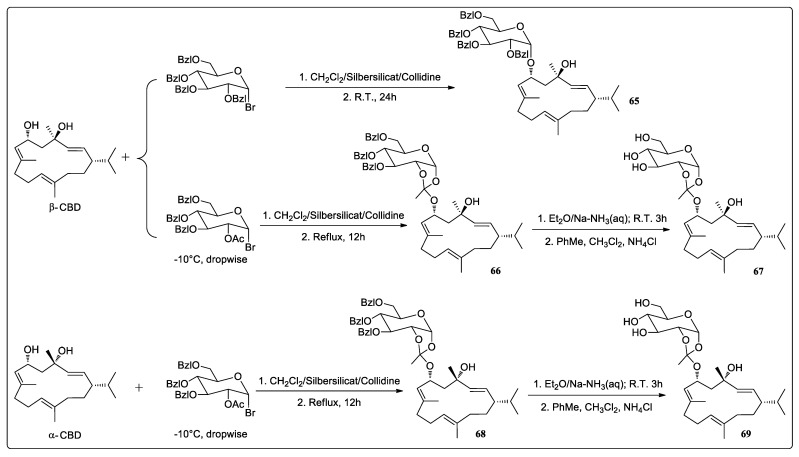
Synthesis of the 4-*O*-pyranoglucosyl derivatives of β-CBD (**65**–**67**) and α-CBD (**68**, **69**).

Carbohydrates have been demonstrated to be involved in a variety of physiological events. Glycosylation modifications have received considerable attention from pharmaceutical chemists for their potential use in designing novel clinical drugs [23]. As both α- and β-CBD are insoluble in water, introducing carbohydrate residues into the parent cembrenediol could enhance the solubility of these compounds. A series of α-D-glucopyranosides of α- and β-CBD (**65**–**69**) have been synthesized. During synthesis, pyranosyl bromides and silver silicate are used as glycosyl donors and catalysts, respectively [24]. The synthetic routes and structures of **65**–**69** are shown in Scheme 3.

**Scheme 4:**
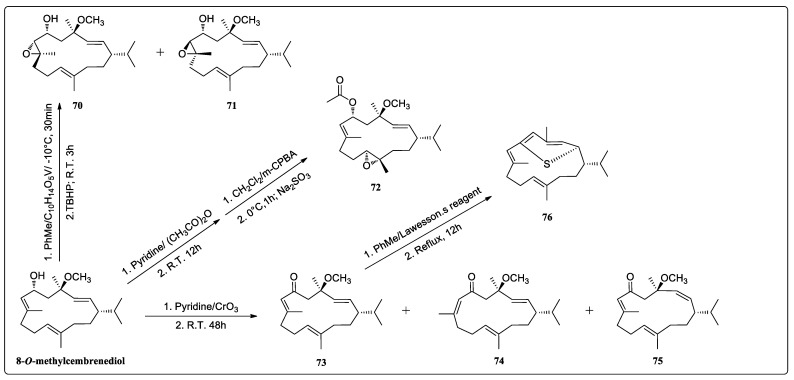
Semisynthetic esterification, oxidation, and epoxidation of 8-*O*-methylcembremediol (**70**–**76**).

8-*O*-Methylcembremediol, a natural product in *N. tabacum*, has been found to display better inhibitory activity on PC-3 prostate cancer cells than β-CBD, and was thus subjected to rational structural modifications. The epoxidized analogs **70** and **71** from 8-*O*-methylcembremediol were prepared by a metal-catalyzed regioselective epoxidation of Δ^7,8^ utilizing t-butyl hydroperoxide and vanadyl acetylacetonate. The acetylated and epoxidized derivative **72** was designed to determine the activity of C-6 acetoxylated (11,12)-epoxy cembranoids. The C6-keto derivatives **73**–**75** were prepared in order to test the importance of the C-6 secondary alcohol moiety with respect to the antimigratory activity and chromic acid oxidation of 8-*O*-methylcembremediol. The reaction of **73** with Lawessonʼs reagent in toluene for 6 h afforded the sulfated derivative **76** [25]. The synthetic routes and structures of **70**–**76** are shown in Scheme 4.

### 3.2. Biotransformation

**Scheme 5:**
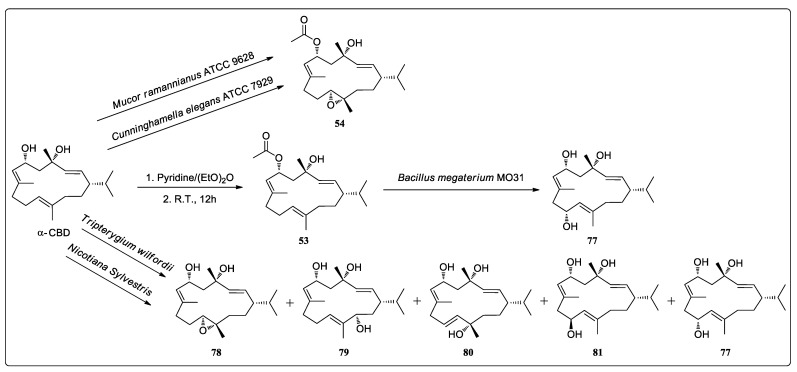
Biocatalytic acetylation, oxidation, and epoxidation of α-CBD (**77**–**81**).

Compared to chemical methods, biological transformations are more advantageous for preparing novel oxygenated derivatives of α- and β-CBD due to their ability to perform stereo- and regioselective reactions; thus, biological transformations can help to increase the bioactivity or decrease the toxicity profiles of cembrenediols as well as support SAR studies. Microbiological conversion of α-CBD using *Mucor ramannianus* ATCC 9628 and *Cunninghamella elegans* ATCC 7929 produced the (10*S*, 11*S*)-epoxidized derivative **54**, while biotransformation of **53** using *Bacillus megaterium* MO31 yielded the C-10 oxidative derivative **77** [19]. In addition, α-CBD can be biotransformed by plant cell cultures that contain *Tripterygium wilfordii* and *Nicotiana sylvestris*, both for varying time periods and by utilizing cells with different ages. For both bioconversions the major product included epoxidized derivative **78**, which was prepared from a regioselective enzymatic attack at the C-11,12 double bond in α-CBD. The minor components included C-10, C-12, and C-13 as well as the enzymatically hydroxylated and rearranged triol derivatives of α-CBD (**77**, and **79**–**81**), which were first reported in species from the genus *Nicotiana* [26]. The synthetic routes and structures of **77**–**81** are shown in Scheme 5.

**Scheme 6:**
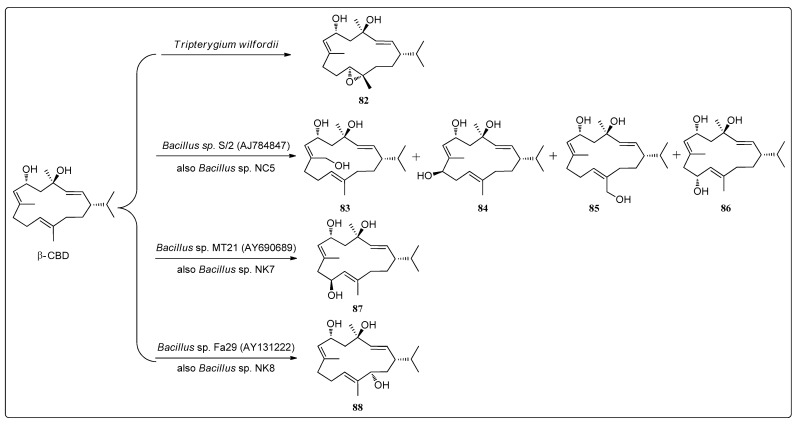
Biocatalytic oxidation and epoxidation of β-CBD (**82**–**88**).

Plant cell cultures with *T. wilfordii* can biotransform β-CBD and achieve enzymatic catalysis at the (11,12)-position, affording the predominant product (11*S*,12*S*)-epoxide analog (**82**), which has been identified on the basis of published spectral data [27]. Thirty growing symbiotic bacterial species from the marine sponge *Negombata magnifica* were assayed for their ability to bioconvert α-CBD. Of these, three *Bacilli* species, NC5, NK8, and NK7, were selected to undergo scaled-up bioconversion. As a result, the biocatalysis of α-CBD was performed using *Bacillus* sp. NC5, producing four hydroxylated metabolites, **83–85**. Fermentation of α-CBD using *Bacillus* sp. NK7 and NK8 produced **87** and **88**, respectively [20]. The synthetic routes and structures of **82**–**88** are shown in Scheme 6.

**Scheme 7:**
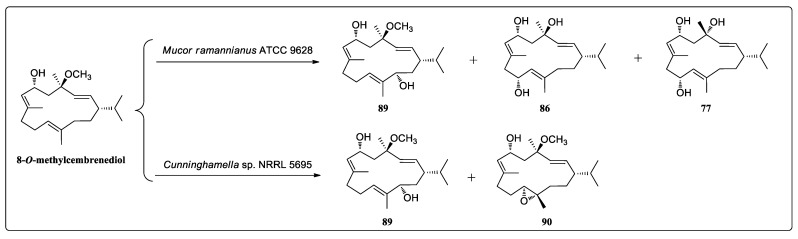
Biocatalytic oxidation and epoxidation of 8-*O*-methylcembremediol (**89**, **90**).

Two fungal strains, *Cunninghamella* sp. NRRL 5695 and *Mucor ramannianus* ATCC 9628, were selected for their ability to biotransform 8-*O*-methylcembremediol into polar metabolites, which was determined through TLC analysis. Scale-up fermentation of 8-*O*-methylcembremediol using *M. ramannianus* ATCC 9628 afforded hydroxylated derivative **89** and two hydroxylated C4-O-demethylated derivatives, S **77** and **86**, while scale-up fermentation of 8-*O*-methylcembremediol using *C.* sp. NRRL 5695 afforded **89** and the (11*S*,12*S*)-epoxidized derivative **90** [25]. The synthetic routes and structures of **89** and **90** are shown in Scheme 7.

## 4. Biological Properties of α-/β-CBD and Its Analogs

Biological studies of α- and β-CBD have mainly focused on their antitumor, neuroprotective, and antimicrobial activities. In this section, their parent, α-/β-CBD, as well as those analogs that exhibit potent biological activities, are discussed.

### 4.1. Antitumor Activity

In 1985, α-CBD and β-CBD were first identified as antitumor agents and found to inhibit tumor promotion in mouse skin [18]. Subsequently, many scholars have conducted wonderful studies on the antitumor properties of α-/β-CBD and its analogs. For example, it was found that α-CBD reduces the activated VEGFR2 levels in various breast cancer cell lines in vitro and reduce the MDA-MB-231 tumor size in mice in vivo. One possible mechanism of action is that α-CBD could downregulate the vasculogenesis marker CD31 and thereby suppress the VEGFR2-paxillin-FAK pathway [28]. In addition, α-CBD could reduce the apoptosis of HepG2 cells through the p53-PUMA, PI3K-Akt, and IL-1-NF-κB-IAP pathways, inhibit cell proliferation, change the permeability of plasma membranes, promote apoptosis, arrest the cell cycle in S phase, and induce apoptosis [29]. In this section, the anticancer properties of α-/β-CBD and its analogs are described using five different levels: extraordinary (IC_50_ < 1 μM or 0.4 μg/mL); significant (IC_50_ 1~10 μM or 0.4~4 μg/mL); moderate (IC_50_ 10~30 μM or 4~12 μg/mL); weak (IC_50_ 30~50 μM or 12~20 μg/mL); and insignificant (IC_50_ > 50 μM or 20 μg/mL).

Previously, α-CBD was chemically modified to enhance its c-Met kinase inhibition and improve the overall antiproliferative cellular potency on MDA-MB-231 and MDA-MB-468 cells [21]. Synthetic analog **3** showed clear enhancement compared to of α-CBD, with IC_50_ values of 19.8 and 22.7 μM, respectively, while **1** was only weakly active, with IC50 values of 38.2 and 39.6 μM, respectively. This revealed that substituting the C6 with a phenyl group could improve the inhibitory activity of c-Met. Both **2** and **4** showed less activity than **3** in the cellular proliferation experiment, indicating that the phenyl moiety is the best substituent at that point for c-Met inhibition. Biological assessment of **5**–**8** revealed that an electron-withdrawing group improved antiproliferative activity, unlike the electron-donating counterpart. Compound **8** exhibited a significant improvement in inhibition compared with **6** and **9**, indicating that the m-position for the chloro substituents on the phenyl ring could enhance the anticancer potency of the compound, while **8** showed c-Met catalytic activity with an IC_50_ value of 3.4 μM. Compared to the chlorinated analogs **6**, **8**, and **9**, the fluorinated analogs **10** and **11** were relatively weak, revealing the importance of substituent hydrophobicity to enhancing activity; this result was further validated by the activities of dichlorinated analogs **12**–**15**. Among them, **12** was the most active, with IC_50_ values of 5.3 and 6.6 μM against MDA-MB-231 and MDA-MB-468 cells, respectively, and inhibited c-Met catalytic activity, with an IC_50_ value of 2.9 μM. Compound **16** inhibited the catalytic activity of c-Met and had an IC_50_ value of 1.8 μM, more potent than compound **17** and nearly comparable to compound **12**. Compound **18** exhibited significant anticancer activity, with IC_50_ values of 1.3 and 2.1 μM, respectively, and potently c-Met inhibitory activity with an IC_50_ value of 1.1 μM. More importantly, **18** possessed excellent in vivo antitumor activity in a breast cancer xenograft athymic mouse model.

The antiproliferative activity of α-CBD and its analogs in highly malignant mouse +SA mammary epithelial cells has been studied as well [19]; α-CBD, **56**, **57**, **63**, and **64** showed moderate activity, with IC_50_ values of 15–40 μM, while *δ*-tocotrienol is a positive drug, with an IC_50_ value of 7 μM. The most active analogs were **56** (13*R*) and **57** (13*S*), with IC_50_ values of 30 and 20 μM, respectively. This clearly indicates the importance of the C13 hydroxy group, especially β-hydroxy, in enhancing this activity. Analogs **19**–**21** were devoid of anticancer activity, revealing the key role of the free C6 hydroxy group, a result supported by the activity of **53**, which was lower than that of α-CBD. The biocatalytic product **77** exhibited no effect against mammary tumor cells, revealing the importance of the free C10 hydroxy group. The inactive effect of **58** and **59** indicates that Δ^11,12^ cannot be substituted by heterocyclic SP2, SP, or heteroatom-containing functional groups. Interestingly, **62** was almost devoid of anticancer activity, while the activity of **63** was greater than that of α-CBD, indicating the potential selectivity of the double-bond geometry. Compound **64** showed more potent anticancer activity than that of its parent α-CBD at higher doses, with an IC_50_ of 15 μM, revealing that the 14-membered macrocycle is not necessary for anticancer bioactivity.

The anticancer activities of β-CBD and its analogs were assayed on PC-3M-CT^+^ cells (human highly metastatic prostate cancer cells) [20]. β-CBD showed little cytotoxic and potential anticancer activities at concentrations of 10–20 nM; however, it was cytotoxic at 50 μM or higher. β-CBD inhibited the basal growth of PC-3M cells and abolished cell proliferation of Calcitonin (CT)-stimulated PC-3M at a certain concentration. Semisynthetic derivatives **50**–**52** displayed potent anticancer effects against PC-3M cells at concentrations from 5 to 50 nM and exhibited lower toxicity than β-CBD at 50 nM. Biocatalytic products **86**–**88** possessed potent anticancer activity at 50 nM without cytotoxicity as compared to β-CBD. The effects of **50**–**52** on baseline and CT-stimulated TER and paracellular permeability were assayed. Only **51** could stabilize junctional complexes and considered worthy of further study.

The anticancer effects of 8-O-methylcembrenediol and its analogs against PC-3M-CT^+^ cells were evaluated [25]. At a concentration of 50 μM, **70**, **73**, **77**, **86**, **89**, and **90** exhibited better activity than that of the vehicle control (DMSO, *p* < 0.05), while **74** was insignificant. These results indicate that the *E*-geometry is the preferable geometry for exertion of an anticancer effect, rather than the Z-geometry of Δ^7,8^. At 50 μM, 8-O-methylcembrenediol and **71** were cytotoxic, and their activity was thus evaluated at concentrations from 10 to 1000 nM. Both showed no activity at the lowest tested dose (10 nM) and exhibited a typical dose–response effect at 100, 500, and 1000 nM versus the vehicle control. A dose of 1000 nM 8-O-methylcembrenediol and **71** reduced the disaggregation and cell migration of PC-3M-CT+ spheroids by 65% and 75%, respectively, while the positive control was inactive in this dose range and only showed activity at 50–200 μM. Based on the above biological results, epoxidation, acetylation, oxidation, or hydroxylation at C6 of 8-O-methylcembrenediol reduced the anticancer effect, while only the (7S,8*S*)-epoxidized derivative **71** showed potent activity. This result indicates the direction of future modifications; specifically, (7*S*,8*S*)-oxirane could be used to replace the *E* Δ^7,8^ system. Additionally, 8-O-methylcembrenediol and **71** were the two most active compounds in the wound-healing assay at a concentration of 50 μM. Compared to β-CBD and the positive control 8-O-methylcembrenediol was more active, indicating that the compound’s effect could be enhanced through the C4-O-methylation of β-CBD. Compounds **73** and **90** were the next most active compounds, indicating that the effects of C6-oxidation on the ketone or (11*S*,12*S*)-epoxidation activity were negligible. The least active compounds were **86** and **89**, indicating that the C-10 or C-13-hydroxylation of β-CBD reduced the activity. The poor activity of **75** indicates that the E geometry, rather than the Z-geometry of Δ^2,3^, is most conducive for improving the activity.

Both α-CBD and its semisynthetic analogs **22**–**44** and **45**–**49** were previously assayed for their anticancer activities against HL-60 (human promyelocytic leukemia cell line), SMMC-772 (human hepatocellular carcinoma cell line), A-549 (human lung cancer cell lines), and MCF-7 (human breast cancer cell line) using MTS assay [22]. At a concentration of 40 μM, the parent nucleus α-CBD exhibited moderate inhibitory activities on tumor cell growth (29%, 25%, 20%, 47%, and 10%), and these activities were parallel to the data reported in the literature. Among the C6-esterified derivatives **22**–**44** most of the compounds showed different degrees of activity on the five cell lines, except for the **22**, **23** and **28** derivatives with a short carbon chain in the carboxyl group. More specifically, compared to a positive control (cisplatin), **24**–**27** and **29**–**32** exhibited competitive inhibitory effects against MCF-7 cells and **27** and **30**–**32** exhibited even better anticancer effects, with IC_50_ values of 16.57, 15.45, 19.11 and 17.42 μM, respectively. Compared to the positive control (cisplatin), compounds **33**–**37**, **39**–**42**, and **44** showed competitive inhibitory effects against SMMC-772 cells; **35** and **41** exhibited the most activity, with IC_50_ values of 13.26 and 11.44 μM, respectively. For the C6 alkylated derivatives **45**–**49**, only **46** and **49** showed weak effects against A-549 cells; the other compounds were inactive. SAR revealed that the phenylacetate substituents at the C6 position play an important role in the anticancer activity of the compounds.

### 4.2. Neuroprotective Activity

The first evidence that cembranoids possessed neuroprotective activity was found in 1981, when it was determined that a marine cembranoid analog (lophotoxin) could affect nicotinic acetylcholine receptors (AChRs) [30,31]. Previously, α- and β-CBD had been evaluated on human α4β2 neuronal AChRs and human α3β4 ganglionic AChRs, with the potency found to be greater for β-CBD than for α-CBD on both receptors. β-CBD was able to completely and noncompetitively inhibit the agonist-induced ^86^Rb flux via the above two receptors, and showed a slightly higher potency on the α3β4 AChR (IC_50_ value, 2.2 μM) than on the α4β2 AChR (IC_50_ value, 19.1 μM) [32]. However, later studies on rat α4β2 neuronal AChRs expressed in *Xenopus* oocytes found no difference in the potencies of α- and β-CBD in inhibiting agonist-induced currents; both compounds could more effectively inhibit the nicotine-induced response than the acetylcholine-induced response, and the inhibition was significantly increased through preincubation with cembranoid [33]. Additionally, at a dose of 6 mg/kg β-CBD could inhibit the expression of sensitization to the level of saline controls in mecamylamine-injected sensitized rats [32]. The effect of tobacco cembranoids on AChRs and related ligand-gated ion channels has been systematically summarized [34], several excellent works have subsequently been published on the topic.

It has been reported that β-CBD can improve the effect of NMDA (N-methyl-D-aspartate)-induced excitotoxicity in vivo. β-CBD protected the function of the hippocampal slice in a dose-dependent manner, with an EC_50_ value of 0.24 μM. Further studies suggested that the neuroprotective effect of β-CBD might be mediated by the activation of the PI3-kinase/Akt antiapoptotic cascade. Thus, GSK3-β (glycogen synthase kinase 3-β) was inhibited, causing the reversal of NMDA-induced apoptosis. The Raf/MEK/ERK cascade does not participate in β-CBD-mediated neuroprotection [35]. β-CBD was able to reverse POX (OP paraoxon) inhibition of population spikes (PSs), with an EC_50_ value of 0.8 μM. While atropine alone could not protect against POX neurotoxicity, it enhanced protection by adding β-CBD. β-CBD could not regenerate AChE (acetylcholinesterase), indicating that the toxic effect of POX on hippocampal function might not be directly related to AChE inhibition [36]. β-CBD improved the neurotoxicity induced by DFP (diisopro-pylfluorophosphate) in hippocampal area CA1, decreasing the number of dead neurons by 50% when injected before or after the application of DFP, and significantly reduced the number of activated astrocytes [37].

The parents α-CBD, β-CBD, 8-O-methylcembrenediol, and their semisynthetic or biocatalytic derivatives **62**, **70**, **72**, **73**, **75**, **76**, **83**–**87** were previously evaluated for their effect on protecting the population spikes from DFP-induced damage and intrinsic toxicity. When β-CBD (10 μM) was applied for 30 min after DFP, an obvious reversal of DFP toxicity from 28% to 86% recovery was recorded (*p* < 0.01). At the same concentration of 10 μM, analogs **62**, **72**, **73**, **75**, **76**, and **83**–**87** exhibited similar effects to those observed with β-CBD; analog **70** resulted in an incomplete reversal of DFP toxicity, while α-CBD and 8-O-methylcembrenediol completely lost their protective activity. SAR analysis revealed that hydroxylation at C-9, C-10, C-19, and C-20 (**83**–**87**) or oxidation of C-6 hydroxyl to a keto group (**62**) maintained the full neuroprotective activity of β-CBD. Despite having an entirely different chemical skeleton from that of other cembrenediols, **76** possessed protective activity as well. It has been demonstrated by a comparison of α-CBD and β-CBD that the 4R configuration is necessary for activity. A free β-hydroxy at C-4 is important for anticancer activity as well, as this activity is lost by the methylation of C-4 hydroxy (as occurs in 8-O-methylcembrenediol). The carbonyl at C-6 (**73** and **75**) can restore the lost protective effect of 8-O-methylcembrenediol. The Δ^2,3^ configuration may not be essential for activity, as suggested by the potent activity of both the E- and Z-oriented Δ^2,3^ in **73** and **75**, respectively. The (11*S*,12*S*)-epoxy functionality could rescue the protective activity of 8-O-methylcembrenediol, as suggested by the activity of its (11,12)-epoxy analogs **72** and **90**. The C6-O-acetate in **72** did not affect the activity of **90**. The (7*R*,8*S*)-epoxy present in **70** partially rescued the activity of 8-O-methylcembrenediol [38].

In addition to the abovementioned protective activities of β-CBD against the neurotoxicity induced by NMDA, POX, and DFP, it was found that β-CBD showed potent neuroprotective activity in a 6-OHDA (6-hydroxydopamine)-induced Parkinson’s disease (PD) rat model. Treatments with β-CBD (6 and 12 mg/kg) significantly improved the outcomes of 6-OHDA-induced PD in vivo, as indicated by reduced forelimb asymmetry scores and corner test scores (*P* < 0.05). The neuroprotective effect of β-CBD was demonstrated by decreased depletion of TH (tyrosine hydroxylase) in the striatum and SN (substantia nigra) on the side injected with 6-OHDA. Moreover, β-CBD protected differentiated neuro-2a cells from 6-OHDA-induced cytotoxicity in vitro. The activation of p-AKT and HAX-1 and the inhibition of caspase-3 and endothelial inflammation are involved in β-CBD-mediated protection against 6-OHDA-induced neurotoxicity [39].

The neuroprotective effect of cembranoids could be mediated by their anti-inflammatory activities [40]; α- and β-CBD have both been found to inhibit prostaglandin synthesis, with a lower IC_50_ value than that of acetylsalicylic acid [41]. The neuroprotective effect of β-CBD against NMDA-induced toxicity has been observed in both C57BL/6J wild-type (WT) and α-7-knockout (α7KO) mice. It was found that hippocampal TNF-α and IL-1β levels were decreased with the application of β-CBD following LPS (lipopolysaccharide) exposure in both strains of mice. β-CBD was able to restore LPS-induced cognitive decline in the long-term NOR test. There was a significant increase in the phosphorylation of STAT3, CREB, and Akt1 after treatment with β-CBD in WT mice following LPS exposure. In α7KO mice, only pAkt levels were significantly increased in the cortex. β-CBD significantly upregulated the mRNA levels of ORM2, GDNF, and C3 following LPS exposure, which plays a role in modulating microglial activation, neuronal survival, and memory [42]. In order to investigate whether the central anti-inflammatory activity of β-CBD is active in the peripheral inflammation that is triggered by brain ischemia, macrophages treated with LPS were used as an inflammatory model and then treatment with β-CBD was performed. The results showed that β-CBD is related to a reduction in NFKB1 and ITGB5 gene expression, increased phosphorylation of NF-kB, and a decrease in macrophage adhesion in a blood–brain barrier model, suggesting that β-CBD can partially modulate the peripheral immune response and might be a potential drug against postischemic inflammation [43].

### 4.3. Antimicrobial Activity

α- and β-CBD were first identified as antimicrobial agents in 1977. These two compounds were assayed for their inhibitory effects on the germination of *Peronospora tabacina* and *Monilinia fructicola*. It was found that the toxicity of β-CBD is greater than that of α-CBD to *P. tabacina*, with EC_50_ values of 17 and 24 μg/mL, respectively, while the reverse situation is true for *M. fructicola*, with EC_50_ values of >100 and 56 μg/mL, respectively, and EC_25_ values of 100 and 40 μg/mL, respectively [44]. Another in vitro microbial bioassay evaluated the influence of these two compounds on the germination and germ tube morphology of *P. tabacina* sporangia. Germination of sporangia was completely inhibited by α- and β-CBD at a concentration of 10 μg/cm^2^. In contrast to a previous report, the activity of α-CBD was greater than that of β-CBD. The toxic effects of α- and β-CBD were not altered by the pH. Diluting the α- and β-CBD to less than 0.1 μg/cm^2^ resulted in a small though significant stimulation of germination. Both α- and β-CBD had previously been identified only as an inhibitor against *P. tabacina* [45]. Later, α- and β-CBD were evaluated for their biological activity against *Colletotrichum lagenarium* (Pass.) Ell. & Halst, the anthracnose pathogen of cucumber, with an IC_50_ value of 6.3 μg/cm^2^ [46].

Both α- and β-CBD have been screened for antibacterial activity against *S. aureus*, *B. subtilis*, *E. coli*, *S. typhimurium*, *Shigella dysenteraie*, *P. aeruginosa*, and *P. vulgaris* as well as for antifungal activities against *Candida albicans*, *Fusarium chlamydosporum*, *Rhizoctonia bataticola*, *Aspergillus niger*, and *Alternaria alternata*. β-CBD showed broad spectrum antimicrobial properties, inhibiting almost all the test bacteria and fungi at a concentration of 50 μg/disc and with a zone of inhibition ranging from 8 to 30 mm, while α-CBD only showed weak activities, with a zone of inhibition less than 30 mm [47].

The antifungal activity of tobacco extracts was evaluated on *Valsa mali*, the causative pathogen of apple tree canker disease, indicating that extracts with a weaker polarity had a higher fungicidal activity and that the inhibitory effect was cultivar-dependent. The effects of flower extracts were higher than those of the leaf extracts. α- and β-CBD were deduced to be the main fungicidal substances able to destroy the endometrial structure of the fungus. At a concentration of 80 μg/mL the cembranoids completely inhibited the growth of *V. mali*, with an EC_50_ value of 13.18 μg/mL. The results indicated that tobacco cembranoids are potential resources for the biological control of *V. mali* [48]. The underlying mechanism of the representative ingredient was further investigated, and the EC_50_ value of α-CBD against *V. mali* was 18.0 μg/mL. Treating *V. mali* with α-CBD led to various mycelial and cellular abnormalities and the up- and downregulation of 94 and 170 differentially expressed genes, respectively; α-CBD substantially altered the expression of the genes involved in the redox process, tetrapyrrole binding, coenzyme binding, heme binding, and iron binding. An enrichment analysis in the Kyoto Encyclopedia of Genes and Genomes revealed significant enrichment of the specific metabolic pathways that involve the set of differentially expressed genes [49].

Both α- and β-CBD can inhibit wild and multiresistant strains of *Botrytis cinerea*, the causative pathogen of crop fungal diseases, with EC_50_ values of 9.67–16.38 μg/mL. At a concentration of 200 μg/mL, α- and β-CBD inhibited *B. cinerea* in different fruits by 71.9 to 84.2%. Significant damage to the membrane system of *B. cinerea* resulted in organelle disintegration while sparing the cytoderm. Investigations of the underlying mechanism revealed that α- and β-CBD increased the activity of chitinase, resulting in an accumulation of N-acetyl-D-glucosamine and causing a significant increase in conductivity and cellular contents by increasing membrane permeability. Furthermore, α- and β-CBD induced membrane lipid peroxidation, which was associated with the oxidative stress reaction. These results provide a scientific foundation for the application of cembrenediols as an alternative biological agent against *B. cinerea* [50]. An investigation of the SAR of tobacco cembranoids and a crude extract with antifungal activities revealed that the antifungal activity of cembranoids was related to the number of hydroxyl groups and double bonds. The existence of Δ^11,12^/Δ^7,8^ and hydroxylation at C-6/C-4 contributed to antifungal activity. Differences in the type and position of substituents affected antifungal activity, of which Δ^11,12^ was the most influential factor. Moreover, the change in substituent configuration at C-4 and C-12 had an insignificant effect on antifungal activity [51].

## 5. Patent Release of α-/β-CBD and Their Analogs

Patents related to the application of α-/β-CBD and their analogs were searched via structural retrieval in the Scifinder^®^ database for the period from 2000 to 2022. After removing duplicates, a total of ten documents that were closely related to this study were obtained; these are summarized in Table 1. The documents contained four granted PCTs, four granted American patents, and two published Chinese patents. The contents of these ten selected patents are consistent with the aforementioned published journal articles, and can be classified into three application fields, namely, the neuroprotective, anticancer and antimicrobial fields.

## 6. Conclusions and Perspectives

This review summarizes a total of 90 semisynthetic and biotransformed analogs of α- CBD and β-CBD reported between the beginning of 1962 and the end of 2022. A research team led by Professor Khalid A. El Sayed (University of Louisiana at Monroe) performed many excellent studies on the antitumor effects and structure–activity relationship of α- and β-CBD and their derivatives. The research team of Professor P.A. Ferchmin (Universidad Central Del Caribe) conducted systematic studies on the neuroprotective activities of β-CBD, while researchers from the Tobacco Research Institute of Chinese Academy of Agricultural Sciences performed a comprehensive study on the antifungal activities of α- and β-cembrenediol. Overall, α-CBD and its derivatives show greater anticancer advantages than β-CBD, while β-CBD has more potent neuroprotective activities than those of α-CBD; both α- and β-CBD possess moderate antimicrobial effects. The more recent research has great value in promoting the application of tobacco cembrenediol. The potential lead compounds that have been derived from α- and β-CBD are listed in Table 2.

Although previous scientific studies have demonstrated that α-CBD and β-CBD and their analogs (which have been registered as patents in many countries) might be potential candidates for the treatment of anticancer and neurodegenerative diseases and even for agricultural diseases, there are several issues that must be overcome before they become drugs. First, α- and β-CBD have poor solubility in water, possibly causing a low absorption and bioavailability. Second, according to our unpublished experimental results, while α- and β-CBD are stable in methanol and ethanol solutions, they become unexpectedly reversed in acetonitrile solutions, and can even be degraded within several minutes. Increasing the stability of α-CBD and β-CBD and their analogs thus represents a great challenge for their development in future new drugs. Additionally, although the intake of cembranoids through tobacco smoking consumption is not desirable (commercial tobacco is poor in cembranoids), this matrix deserves to be studied for the presence of these bioactive compounds.

## Figures and Tables

**Table 1:** Published and granted patents related to α- and β-CBD and their semisynthetic analogs.

Patent Title	Application Fields	Patent No.	Ref.
Neuronal circuit-dependent neuroprotection by interaction between nicotinic receptors	Neuroprotective	WO2008/002594A2	[52]
Methods and compositions for protecting and treating neuroinjury	Neuroprotective	WO2011/008585	[53]
Therapeutic application of cembranoids against HIV virus replication, HIV-associated neurocognitive disorders and HIV virus-induced inflammation	Neuroprotective	US8835512B2	[54]
Tobacco cembranoids block the expression of the behavioral sensitization to nicotine and inhibit neuronal acetylcholine receptors	Neuroprotective	US6489357B1	[55]
Therapeutics and methods to treat angiogenesis related pathologies	Anticancer	WO2018/213824A2	[56]
Antineoplastic agent	Anticancer	WO86/02835	[57]
Anticancer tobacco cembranoids	Anticancer	US79777384B1	[58]
Antitumor agent	Anticancer	US4701570	[59]
Preparation method and application of cebatrien-4-ol-6-carboxylate	Anticancer	CN109970558A	[60]
Method for extracting cembrane diterpenes from tobacco inflorescence	Antimicrobial	CN105001052A	[61]

**Table 2:** Potential lead compounds derived from α-CBD and β-CBD.

NO.	Chemical Structures	Potent Bioactivities (IC_50_ or EC_50_ Value)	Ref.
**α-CBD**	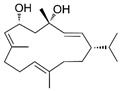	Anti-proliferation on MDA-MB-231 (34.3 μM), MDA-MB-468 (39.3 μM), MCF-7 (61.2 μM), T-47D (61.9μM), SK-BR-3 (48.9 μM), and BT-474 (52.6 μM) cell lines; Antifungal effect against *P. tabacina* (24 μg/mL), *V. mali* (18.0 μg/mL), and *B. cinerea* (9.67–16.38 μg/mL);	[21,22,28,44,45]
**β-CBD**	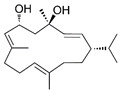	Protective against neurotoxicity induced by NMDA (0.24 μM), POX (0.8 μM), and DFP in rat hippocampal slices; Protective activities on 6-OHDA-induced PD model; Antifungal effect against *P. tabacina* (17 μg/mL), *V. mali*, and *B. cinerea* (9.67–16.38 μg/mL);	[35,36,37,39,44,45]
**8**	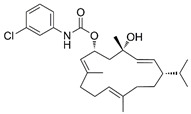	Anti-proliferation on MDA-MB-231 (9.1 μM) and MDA-MB-468 cells (10.6 μM); Inhibition on c-Met catalytic activity (3.4 μM);	[21]
**12**	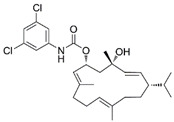	Anti-proliferation on MDA-MB-231 (5.3 μM) and MDA-MB-468 cells (6.6 μM); Inhibition on c-Met catalytic activity (2.9 μM);	[21]
**16**	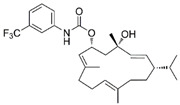	Anti-proliferation on MDA-MB-231 (4.9 μM) and MDA-MB-468 cells (5.8 μM); Inhibition on c-Met catalytic activity (1.8 μM);	[21]
**18**	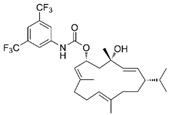	Anti-proliferation on MDA-MB-231 (1.3 μM), MDA-MB-468 (2.1 μM), MCF-7 (7.2 μM), T-47D (11.2 μM), SK-BR-3 (4.7 μM), and BT-474 (3.9 μM) cell lines; Inhibition on c-Met catalytic activity (1.1 μM);	[21,22]

## Data Availability

All data is contained within the article.
